# Phenotype, function and T cell receptor repertoire of tumor-infiltrating lymphocytes in patients with pancreatic ductal adenocarcinoma

**DOI:** 10.1186/2051-1426-3-S2-P44

**Published:** 2015-11-04

**Authors:** Isabel Poschke, Michael Flossdorf, Marta Faryna, Frank Bergmann, Jessica Hassel, Oliver Strobel, Rienk Offringa

**Affiliations:** 1 German Cancer Research Center (DKFZ), Heidelberg, Germany; 2 3BioNTech Diagnostics GmBH, Mainz, Germany; 3 Heidelberg University Hospital, Heidelberg, Germany

## Background

Pancreatic ductal adenocarcinoma (PDAC) is a devastating disease with a median survival of only about two years even in the 20% of patients that present early enough to be eligible for surgical resection and adjuvant chemotherapy. With this urgent medical need in mind, we are exploring the use of adoptive T cell transfer in patients with PDAC, as this therapy has shown remarkable clinical success in patients with advanced melanoma.

## Results

Despite the notion that, different from melanoma, PDAC is a non-immunogenic tumor, we observe at least moderate (>150 CD3^+^cells/mm^2^) T cell infiltration in 60% of patient biopsies analyzed by immunohistochemistry (n=66). Flow cytometric analysis shows that tumor-infiltrating T lymphocytes (TILs) predominantly display an activated effector memory phenotype with signs of exhaustion or prolonged antigen exposure, such as high PD1 (n≥30).

Upon *in vitro* culture TILs can be expanded from 85% of PDAC patients (n=96) and show growth capacity and phenotypes similar to melanoma patient TIL (n=62). The majority of tested PDAC TIL cultures produce IFN-γ in response to autologous tumor cells in an MHC-I dependent manner, but cross-reactivity and a low response magnitude are common observations.

In order to gain insight into the original tumor-reactivity of TILs before expansion, we studied the T cell receptor (TCR) repertoire by deep sequencing and made the following observations: the TIL TCR repertoire is i) distinct from the broad repertoire observed in the blood; ii) usually dominated by large T cell clones, possibly due to *in situ* expansion after tumor-antigen encounter; and iii) in most patients not maintained during the TIL expansion period, likely leading to a loss of important T cell clones and a shift in tumor-reactivity in the TILs available for patient treatment after *in vitro* expansion.

Deep sequencing of the TCR repertoire in combination with TCR cloning provides us with a tool to study TIL reactivity directly *ex vivo* and to ‘rescue’ TIL-TCRs with valuable reactivities that could be reintroduced in the form of genetically engineered T cells. Whether PDAC TILs are reactive towards shared antigens and/or mutation-derived neo-antigens is currently being investigated based on exome and RNA sequencing data from a subset of patients.

